# Real‐World Data on the diagnosis of Alzheimer’s Disease in routine clinical practice: Insights from the Australian Dementia Network “ADNeT” Registry

**DOI:** 10.1002/alz.085008

**Published:** 2025-01-09

**Authors:** Stephanie Alison Ward, Xiaoping Lin, Kasey Wallis, Henry Brodaty, Christopher C. Rowe, Colin L. Masters, Sharon L Naismith, Susannah Ahern

**Affiliations:** ^1^ University of New South Wales, Sydney, NSW Australia; ^2^ Monash University, Melbourne, VIC Australia; ^3^ Prince of Wales Hospital, Sydney, NSW Australia; ^4^ Australian Dementia Network (ADNeT), Melbourne Australia; ^5^ Austin Health, Heidelberg, VIC Australia; ^6^ Florey Institute of Neuroscience and Mental Health, Parkville, VIC Australia; ^7^ The University of Sydney, Sydney, NSW Australia

## Abstract

Real‐world data on the uptake, effectiveness and safety of new diagnostics and disease‐modifying (DMT) treatments for Alzheimer’s Disease (AD) are imperative. This can be achieved through patient registries. A major challenge is how to embed registry data capture into routine clinical practice. To optimize coverage and maintain data contribution over time, registry participation needs to create maximal benefits, yet impose minimal burdens, on clinical sites.

In the experience of the Australian Dementia Network (ADNeT) Registry, it has been the unique insights that clinicians obtain into their own diagnostic practices that has incentivised ongoing participation. The ADNeT Registry collects data primarily to measure the quality of diagnosis and clinical care provided to people with either mild cognitive impairment or dementia and was pragmatically designed to be utilized in multiple diagnostic settings and by different clinical craft groups. Clinicians enter a brief minimum dataset, based upon routine clinical data, at the time of diagnosis. These data are augmented by the ADNeT Registry’s collection of patients and/or carer‐reported experience and outcome measures and will be further augmented by planned linkage with administrative datasets.

By January 2024, the ADNeT Registry had collected data on over 4000 participants, from 66 sites, had issued 4 benchmarked site reports, and published 2 annual reports. Of this cohort, 75% of people with dementia had an AD subtype. At the national level, considerable variation exists in diagnostic time intervals, and in the use of functional neuroimaging and biomarkers. At the individual site level, clinicians report regularly comparing their individual data against Registry benchmarked data to review, and if some cases, improve, their diagnostic approaches.

The Registry’s explicit focus on supporting clinicians is further enhanced by broader achievements of ADNeT, including the publication of memory clinic guidelines, streamlined processes for accessing clinical trials, and appropriate use recommendations for DMTs. Collectively, ADNeT and the ADNeT Registry provides a strong foundation for additional data capture on DMT prescription, safety, and outcome measures, with a design that helps ensure that collected data are truly reflective of the “real‐world”.

